# New perspectives in hepatocellular carcinoma surveillance after hepatitis C virus eradication

**DOI:** 10.1093/gastro/goae085

**Published:** 2024-09-23

**Authors:** Calvin Q Pan, Andrew J Park, James S Park

**Affiliations:** Center for Liver Diseases, Guangzhou Medical Research Institute of Infectious Diseases, Guangzhou Eighth People’s Hospital, Guangzhou Medical University, Guangdong, P.R. China; Division of Gastroenterology and Hepatology, Department of Medicine, NYU Grossman School of Medicine, New York, NY, USA; Division of Hepatology, Department of Medicine, North Shore University Hospital and Donald Barbara Zucker School of Medicine at Hofstra/Northwell Health, Manhasset, NY, USA; Northwell Center for Liver Disease and Transplantation, Northwell Transplant Institute, Northwell Health, Manhasset, NY, USA; Feinstein Visiting Research Scholar Program, Feinstein Institutes for Medical Research, Manhasset, NY, USA; Division of Hepatology, Department of Medicine, North Shore University Hospital and Donald Barbara Zucker School of Medicine at Hofstra/Northwell Health, Manhasset, NY, USA; Northwell Center for Liver Disease and Transplantation, Northwell Transplant Institute, Northwell Health, Manhasset, NY, USA

**Keywords:** liver cancer surveillance, hepatitis C virus, chronic HCV infection, direct-acting antivirals, sustained virologic response, advanced fibrosis

## Abstract

Achieving a sustained virologic response (SVR) through direct-acting antivirals for hepatitis C virus (HCV) infection significantly reduces the long-term risk of hepatocellular carcinoma (HCC), particularly in patients with advanced fibrosis (F3) or cirrhosis (F4). However, despite this improvement, the risks associated with HCC and the optimal surveillance strategies for patients who have achieved SVR remain topics of debate. This controversy is compounded by challenges in reliably staging liver fibrosis non-invasively, especially at advanced fibrosis (F3), and the unclear cost-effectiveness, modality, frequency, and duration of HCC surveillance in individuals with SVR but without cirrhosis. These factors contribute to significant variations in surveillance guidelines recommended by different professional societies. Therefore, there is a pressing need for an optimal surveillance strategy that is both simplified and cost-effective to facilitate wider adoption by clinicians. This review article evaluates the existing data, addresses ongoing controversies, and aims to provide new perspectives on HCC surveillance strategies for patients who have achieved SVR from HCV.

## Introduction

Hepatitis C virus (HCV) infection remains a significant global health threat, strongly associated with elevated morbidity and mortality rates, particularly due to hepatocellular carcinoma (HCC) worldwide. HCV, a small, enveloped, positive-sense single-stranded RNA virus belonging to the family Flaviviridae, does not integrate into the human genome to directly induce hepatocarcinogenesis. Instead, approximately 90% of HCC cases attributed to HCV are preceded by cirrhosis, a condition resulting from long-term liver damage and inflammation [1]. The remaining cases, comprising less than 10%, may be induced by advanced fibrosis without progression to cirrhosis, influenced by several cofactors. These include HCV genotype 3, prolonged infection duration, co-infections such as hepatitis B virus (HBV) or human immunodeficiency virus (HIV), as well as lifestyle factors such as alcohol consumption, obesity, and metabolic syndrome [2–5].

The introduction of direct-acting antivirals (DAAs) has substantially elevated cure rates for HCV, with rates exceeding 95% [4]. Nevertheless, the risks associated with HCC and the optimal surveillance strategies for individuals who achieve sustained virologic response (SVR) remain subjects of ongoing debate, particularly for patients with advanced fibrosis prior to HCV treatment. Additionally, the extent to which the risk of HCC is reduced in patients experiencing regression of hepatic fibrosis post-HCV eradication is not fully elucidated [6, 7]. Given these complexities, there is a compelling need to comprehensively evaluate current data and develop updated perspectives on HCC surveillance strategies following HCV eradication, particularly for those with prior advanced fibrosis or cirrhosis. This review aims to address these critical issues and propose guidelines for effective HCC surveillance tailored to the specific risk profiles of patients post-SVR.

## Effect of HCV eradication in reducing disease progression and HCC

### Improvement in liver function

Achieving a SVR through DAAs for HCV confers numerous long-term health benefits, particularly in improving liver function. This improvement is evidenced by reductions in Child-Pugh-Turcotte and Model for End-Stage Liver Disease (MELD) scores, which are critical indicators of liver disease severity. Furthermore, SVR is associated with a decreased risk of complications related to cirrhosis, such as hepatic decompensation, and a significant reduction in liver-related mortality [8, 9].

In addition to these improvements, the eradication of HCV has been shown to substantially reduce the risk of HCC in patients with advanced liver disease, although this risk is not entirely eliminated. Studies have demonstrated that the incidence of HCC decreases significantly following SVR, particularly in patients with mild to moderate fibrosis. However, patients with advanced fibrosis or cirrhosis prior to HCV treatment remain at risk, necessitating ongoing surveillance [10, 11]. Given the profound impact of HCV eradication on liver function and disease progression, it is critical to continue monitoring patients post-SVR, particularly those with pre-existing advanced fibrosis or cirrhosis, to mitigate the residual risk of HCC and other complications.

### Reduction in HCC risk

In the evaluation of the Hepatitis C Antiviral Long-Term Treatment Against Cirrhosis (HALT-C) cohort for the incidence of HCC and associated risk factors, the cumulative 5-year incidence of HCC was comparable between peginterferon-treated patients and controls (5.4% vs 5.0%, *P *=* *0.78). Additionally, HCC incidence was notably higher in patients with cirrhosis than in those with bridging fibrosis (7.0% vs 4.1%, *P *=* *0.08) [12]. Previous studies have shown that achieving SVR with DAAs significantly reduces the long-term risk of HCC, particularly in patients with advanced fibrosis (F3) or cirrhosis (F4), though it does not completely eliminate this risk [13, 14].

This risk reduction is evident in a retrospective analysis from the Veterans Affairs (VA) system, which estimated HCC occurrence with a mean follow-up of 6.1 years [13]. The incidence of HCC was highest in patients with cirrhosis and treatment failure (3.25 per 100 patient-years [/100 PY]), followed by those with cirrhosis and SVR (1.97/100 PY), no cirrhosis and treatment failure (0.87/100 PY), and no cirrhosis and SVR (0.24/100 PY). The study reported an overall 71% lower risk of HCC in patients who achieved SVR compared to those who did not. Importantly, treatment with DAAs was not associated with an increased risk of HCC compared to previous interferon-based therapies [13].

Further evidence from another VA study by Kanwal *et al.* [10] reinforced these findings. This retrospective analysis of 22,500 patients treated with DAAs (19,518 achieving SVR and 2,982 without SVR) demonstrated a 76% reduction in the risk of HCC among those who achieved SVR compared to those who did not. Compared with patients without SVR, those with SVR had a significantly reduced risk of HCC (0.90 vs 3.45 /100 PY; adjusted hazard ratio [HR], 0.28; 95% CI, 0.22–0.36). Patients with cirrhosis had the highest annual incidence of HCC after SVR (1.82 vs 0.34 /100 PY in those without cirrhosis; HR, 4.73; 95% CI, 3.34–6.68). There was no association between DAAs and HCC. The risk of HCC remained high in patients with cirrhosis even after achieving SVR, underscoring the importance of ongoing HCC surveillance [10].

These data were further corroborated by a prospective analysis of 9,895 French patients in the ANRS CO22 Hepather cohort, where 7,344 were treated with DAAs and followed for a mean duration of 33.4 months. This study confirmed a significant reduction in the risk of HCC among those who achieved SVR, with an adjusted HR of 0.66 (95% CI, 0.46–0.93), demonstrating that DAA treatment effectively reduces HCC risk, though the risk is not entirely eliminated [14].

Additionally, a study by Calvaruso *et al.* [15] provided further insights into the impact of DAA-induced SVR on HCC incidence. In a prospective study involving 2,249 patients with HCV-related cirrhosis, 2,140 patients (95.2%) achieved SVR following DAA treatment, with 95.9% in Child-Pugh class A and 88.3% in Child-Pugh class B (*P *<* *0.001). Over a mean follow-up period of 14 months, 78 patients (3.5%) developed HCC, highlighting the efficacy of SVR in reducing liver cancer risk. At 1-year post-DAA treatment, the incidence of HCC was 2.1% in patients with Child-Pugh class A who achieved SVR, compared to 6.6% in those who did not. Among patients with more advanced liver disease (Child-Pugh class B), HCC incidence was 7.8% in those who achieved SVR vs 12.4% in those who did not achieve SVR (*P *<* *0.001) [15]. These findings collectively underscore the necessity of continued surveillance in patients with advanced liver disease, even after achieving viral clearance, due to their ongoing, albeit reduced, risk of HCC.

### Regression of hepatic fibrosis

The regression of liver fibrosis following SVR after HCV cure has been a subject of growing interest but remains incompletely characterized, largely due to the decreased reliance on liver biopsies post-SVR. The variability in fibrosis regression is influenced by several factors, including the initial severity of fibrosis, the distribution of fibrotic tissue, ongoing environmental and genetic factors, and the presence of comorbid conditions such as obesity, metabolic syndrome, and alcohol use that can continue to drive fibrosis progression even after viral eradication [7]. In recent years, non-invasive diagnostic tools like transient elastography (TE), magnetic resonance elastography, and serum biomarkers have become essential in assessing fibrosis regression, offering a safer and more practical alternative to liver biopsies [7].

A single-center prospective study by Abu-Freha *et al.* [16], involving 209 patients with a mean follow-up time of 4.5 ± 1.3 years post-DAA treatment, found that 57% of patients demonstrated improvement in fibrosis, 7% experienced fibrosis progression, and 36% showed no change. Among patients with liver stages of F3/F4, 28% regressed to moderate fibrosis (F2 or less) based on liver stiffness measurements (LSM). These findings underscore the potential for significant fibrosis regression following DAA therapy but also highlight that a substantial proportion of patients either exhibit no change or experience worsening fibrosis. This variability suggests that even after achieving SVR, ongoing monitoring and management of comorbidities are crucial to prevent further liver damage and complications.

Supporting these findings, a systematic review and meta-analysis by Singh *et al.* [17] indicated that liver stiffness, as measured by TE, is a strong predictor of clinical outcomes, including the risk of decompensated cirrhosis and HCC. The study highlighted that patients with persistent high liver stiffness values post-SVR remain at increased risk for adverse outcomes, emphasizing the need for continued surveillance, particularly in those with advanced liver disease. Moreover, the inconsistency in fibrosis regression post-SVR may explain the variable reduction in HCC risk observed across different patient populations. While SVR significantly reduces the overall risk of HCC, patients with persistent fibrosis or other risk factors—such as metabolic syndrome, ongoing alcohol use, or co-infections—may continue to have an elevated residual risk [11]. This underscores the necessity for tailored follow-up strategies and interventions to manage these ongoing risks, even after successful antiviral treatment.

## Hepatic fibrosis and HCC risk before and after DAA therapy

Accurately assessing the extent of hepatic fibrosis before initiating HCV treatment is crucial, particularly given the increased risk of HCC associated with advanced fibrosis and cirrhosis prior to achieving SVR. The American Association for the Study of Liver Diseases-Infectious Disease Society of America (AASLD-IDSA) 2023 updates recommends using a combination of non-invasive methods to assess liver fibrosis before starting DAAs [18]. TE, which measures liver stiffness, has been extensively validated and is widely utilized for staging fibrosis in patients with HCV, offering a reliable correlation with histological stages of fibrosis [19]. In contrast, the fibrosis index based on the four factors (FIB-4) index is valued for its simplicity, cost-effectiveness, and ease of use, especially in resource-limited settings where access to more advanced diagnostic tools may be restricted. The FIB-4 index, which combines routine laboratory values—aspartate aminotransferase (AST) and alanine aminotransferase (ALT) levels, platelet count, and patient age—has shown high accuracy in predicting significant fibrosis and cirrhosis. With a cutoff of <1.45, the validation set showed a 90% negative predictive value and 70% sensitivity for excluding fibrosis. A score above 3.25 had a 65% positive predictive value and 97% specificity, strongly suggesting advanced fibrosis or cirrhosis and potentially reducing the need for liver biopsy [20].

Ioannou *et al.* [21] demonstrated an important role of the FIB-4 score in assessing HCC risk before and after achieving SVR. Among patients with cirrhosis before DAA treatment (*n* = 9,784), those with pre-SVR FIB-4 scores ≥3.25 had a higher annual incidence of HCC (3.66%/year) than those with FIB-4 scores <3.25 (1.16%/year). Notably, for DAA-treated patients with cirrhosis and FIB-4 scores ≥3.25, the annual HCC risk decreased from 3.8%/year in the first year after achieving SVR to 2.4%/year by the fourth year (*P* = 0.01). However, patients without cirrhosis before treatment (*n* = 38,351) generally had a low risk of HCC, except for those with pre-SVR FIB-4 scores ≥3.25 (HCC risk, 1.22%/year) and post-SVR FIB-4 scores ≥3.25 (HCC risk, 2.39%/year)—indicating that the risk remained elevated for many years post-SVR.

Cirrhosis can also be identified through non-invasive methods such as TE, where a LSM exceeding 12.5 kPa typically indicates cirrhosis. Other indicators include a FIB-4 score greater than 3.25, liver biopsy findings, a platelet count below 150,000/mm³, or imaging findings such as liver nodularity or splenomegaly [22]. These diagnostic criteria are not only pivotal in guiding HCV treatment decisions but also in determining the appropriate strategy for ongoing HCC surveillance after achieving SVR. While liver biopsy remains the gold standard for staging liver fibrosis, non-invasive methods are increasingly preferred due to their safety, patient comfort, and ability to provide continuous monitoring.

Emerging data underscore the importance of LSM in predicting HCC occurrence in HCV patients treated with DAAs, highlighting its role in ongoing patient management. A systematic review and meta-analysis by You *et al.* [23] found that higher LSM values before and after DAA therapy were significantly associated with increased HCC risk. This correlation suggests that even after achieving SVR, patients with elevated LSM may require more intensive surveillance due to their heightened risk of HCC. Supporting these findings, Ogasawara *et al.* [24] assessed LSM in 398 patients who achieved SVR, evaluating LSM levels before and after achieving SVR and correlating these with the risks of HCC and hepatic decompensation. They defined liver cirrhosis as LSM ≥12 kPa and chronic hepatitis as LSM <12 kPa. Their findings indicated an annual occurrence rate of HCC of 1.5% during the first four years after achieving SVR, with LSM values typically decreasing after DAA treatment but remaining higher in patients who developed HCC than in those who did not, both prior to and following treatment. Multivariate analysis identified LSM and alpha-fetoprotein (AFP) levels at baseline, as well as LSM at SVR at 24 weeks after the completion of HCV DAAs, as significant independent predictors of HCC development.

## HCC risk from co-existing conditions and baseline fibrosis

The risk of HCC in patients who have achieved a cure for HCV is multifaceted, with the presence of co-existing conditions significantly influencing outcomes. Metabolic conditions such as obesity and diabetes, along with heavy alcohol use, are key factors that elevate the risk of HCC even after SVR [25, 26]. In a single-center longitudinal study, Degasperi *et al.* [25] followed 565 HCV cirrhotic patients treated with DAAs over three years, demonstrating that HCC risk was independently associated with factors such as male gender, LSM, FIB-4 scores, and particularly the presence of metabolic conditions.

For patients who achieve SVR with mild or no fibrosis, the persistence of HCC risk is largely driven by non-viral factors such as advanced age, alcohol consumption, and metabolic conditions. El-Serag *et al.* [26] found that even after viral eradication, older patients and those with metabolic conditions or a history of alcohol use remained at elevated risk for HCC. Similarly, a 2018 study by Nahon *et al.* [27] involving 1,270 HCV patients with cirrhosis under surveillance programs highlighted that post-SVR HCC development was often linked to underlying metabolic disorders, underscoring the need for continuous risk management in these populations.

In patients without cirrhosis, less than 10% of HCC cases are attributed to a combination of factors including HCV genotype 3, prolonged infection duration, co-infections such as HBV or HIV, and lifestyle factors like obesity, diabetes, alcohol consumption, male gender, and tobacco use [3–5]. The synergistic interaction of these factors can significantly exacerbate HCC risk beyond what might be expected if each factor acted independently [28].

These findings emphasize the necessity of developing refined HCC surveillance strategies that are both nuanced and adaptable to the evolving understanding of HCC risk factors. Surveillance programs should not only focus on early detection of HCC but also proactively manage modifiable risk factors to mitigate the overall burden of HCC in this vulnerable population.

## Current challenges in HCC surveillance after SVR

Cirrhosis remains a critical risk factor for HCC development after SVR, with the risk nearly 4.7-fold higher in patients diagnosed with cirrhosis than in those without [10]. There is consensus among professional societies that patients with cirrhosis should undergo ongoing HCC surveillance, using a 6-month ultrasound scan (US) with or without AFP testing, after achieving SVR [29–32]. This is because the risk of HCC persists long after the virus is eradicated.

However, there is a lack of consensus for patients with advanced fibrosis (F3) or even early fibrosis (F0–F2). This reflects the persistent yet reduced risk of HCC in these groups. The surveillance strategy for patients with different degrees of fibrosis, especially those with F3, remains a subject of debate due to non-invasive staging challenges and their relatively lower risk of HCC compared with those with cirrhosis [13]. Additionally, there is ongoing debate among professional societies regarding HCC surveillance for patients with different stages of fibrosis other than cirrhosis after SVR. Some guidelines advocate for continued surveillance in patients with advanced fibrosis, highlighting the necessity for a tailored approach based on individual risk assessments.

Patients with chronic HCV infection and advanced fibrosis (F3) are also at increased risk of HCC, as the transition between advanced fibrosis and cirrhosis cannot always be accurately determined using non-invasive methods like TE. Patients with F3 fibrosis present significant challenges in reliable staging through non-invasive methods and exhibit a lower risk of HCC than those with cirrhosis. This complicates decisions around HCC surveillance, as the risk in these patients, although reduced compared with those with cirrhosis, is still present. A critical issue in managing all patients who achieve SVR is the reliable estimation of HCC risk, as this risk is a key determinant of the cost-effectiveness of screening protocols. This challenge has led the European Association for the Study of the Liver (EASL) to recommend semiannual surveillance for patients with advanced fibrosis (F3) or cirrhosis (F4), even after achieving SVR with DAA treatment [29].

The AASLD guidelines suggest refraining from routine surveillance in patients with cirrhosis classified as Child’s class C unless they are on the transplant waiting list, due to their generally low anticipated survival rates [30]. Conversely, the EASL endorses ongoing surveillance for patients with advanced fibrosis (F3), reflecting a more cautious approach toward this high-risk group [29]. The International Liver Cancer Association (ILCA) also recommends ultrasound surveillance not only for those with cirrhosis but also for patients with F3 fibrosis and high GALAD scores, which include gender, age, lens culinaris agglutinin-reactive AFP isoform (AFP-L3), AFP, and des-gamma-carboxyprothrombin (DCP) [31].

Regarding the Asian Pacific Association for the Study of the Liver (APASL), it provides some of the most aggressive recommendations. The APASL advocates for HCC surveillance every 6 months for all patients with HCV before treatment and for the first two years after SVR, irrespective of fibrosis stage. Following this, the APASL recommends annual surveillance for patients with F0–F2 fibrosis stages and semi-annual surveillance for those with F3–F4 fibrosis. Additionally, patients with HCV who also suffer from alcohol abuse and/or diabetes are advised to continue under US surveillance [32]. These society guidelines aim to more accurately predict HCC risk after DAA treatment, enabling a more targeted surveillance approach. The similarities and differences in the professional guidelines in HCC surveillance after SVR are listed in Table 1. The indications, frequency, and optimal duration of HCC surveillance following HCV cure remain subjects of ongoing research and debate, necessitating long-term data on patients with pre-treatment advanced fibrosis or cirrhosis.

## Other perspectives on HCC surveillance

The ongoing debate over the most effective surveillance intervals and modalities underscores the need for further research to optimize HCC surveillance strategies, particularly for patients with F3 fibrosis or those without cirrhosis, where the benefits of routine screening must be carefully weighed against the potential costs and harms after achieving SVR. In the absence of randomized controlled trials addressing HCC surveillance in HCV, modeling studies are crucial for evaluating the efficacy and cost-effectiveness of HCC surveillance [33]. Surveillance for HCC among patients with HCV cirrhosis has been demonstrated to be cost-effective using either semiannual AFP and annual US or triple-phase computed tomography (CT) compared to no surveillance, with the cost of surveillance being less than $50,000 per quality-adjusted life year [34–36]. This cost-effectiveness is comparable to other widely used screening strategies, such as colonoscopy and mammography. An annual HCC incidence of about 1.5% makes surveillance both effective in terms of life-years saved and cost-effective [34].

The sensitivity and specificity of surveillance modalities, including AFP, US, CT, and magnetic resonance imaging (MRI), vary, necessitating tailored surveillance strategies based on individual cases. The suboptimal accuracy of US, particularly in obese patients, highlights the need for improved screening strategies. Contrast-enhanced multiphasic CT and MRI offer higher sensitivity for early-stage HCC detection but are limited by higher costs and potential adverse effects [37, 38]. Kanda *et al.* [32] suggest different surveillance intervals depending on liver fibrosis stage and HCC history. Recent studies, however, suggest that the lower incidence of HCC in F3 patients may not justify the cost of frequent surveillance, as the overall annual incidence is less than 0.5% after SVR [39]. Lockart *et al.* [39] showed in their meta-analysis that the incidence of HCC was 2.1 (95% CI, 1.9–2.4) per 100 person-years among patients with cirrhosis and 0.5 (95% CI, 0.3–0.7) per 100 person-years among patients with F3 fibrosis. Although the incidence of HCC among F3 patients is low, the cost-effectiveness of annual ultrasound screening, which is relatively inexpensive, remains debatable.

Recent advancements in HCC surveillance underscore the evolving role of LSM measured by TE in monitoring fibrosis regression after achieving SVR following DAA therapy. Studies such as those conducted by Semmler *et al.* [40] highlight that LSM, when combined with personal variables, can significantly stratify patients based on their HCC risk. Although significant regression in fibrosis was observed after achieving SVR from DAA therapy, a considerable number of patients still exhibited no change or progression [16]. A meta-analysis summarized by You *et al.* [23] confirms its utility in predicting HCC risk among patients post-SVR. Furthermore, Ogasawara *et al.* [24] establish significant thresholds for LSM, distinguishing between cirrhosis (LSM ≥12 kPa) and chronic hepatitis (LSM <12 kPa), with an observed annual HCC rate of 1.5% over four years. Notably, LSM values tend to decrease following DAA therapy, yet they remain higher in patients with HCC than in those without, both prior to and post-treatment, underscoring its predictive significance [24].

Although the decrease in HCC risk observed over time is likely related to the slow process of liver fibrosis regression following HCV eradication, further study is needed to determine whether these patients can discontinue HCC surveillance [39]. Ioannou *et al.* [21] demonstrated that patients with FIB-4 scores ≥3.25 before and after SVR had the highest HCC risk, whreas those with scores <3.25 before and after SVR had the lowest. Among cirrhotic patients treated with DAAs (*n* = 9,784), those with pre-SVR FIB-4 scores ≥3.25 had a higher annual HCC incidence (3.66% vs 1.16%). The HCC risk in this group decreased from 3.8% in the first year post-SVR to 2.4% by the fourth year (*P* = 0.01). A decrease in FIB-4 scores from ≥3.25 to <3.25 post-SVR was associated with a 50% lower HCC risk, indicating an intermediate risk. This pattern persisted even in patients without pre-treatment cirrhosis, where a high FIB-4 score ≥3.25 after SVR correlated with a significant HCC risk (>2% per year) up to 10 years post-SVR.

## Simplified and risk-stratified HCC surveillance post-DAA therapy

The majority of HCC cases among HCV-infected patients treated with DAAs occur in those with cirrhosis, with an incidence rate greater than 1.5% per year. This rate justifies the cost-effectiveness of surveillance in this population, as studies have shown that surveillance is cost-effective when the annual HCC incidence exceeds 1.5% [34]. In contrast, patients with F3 fibrosis experience an annual HCC incidence of ∼0.5%, which is below the established threshold for cost-effective surveillance [39]. However, patients with additional risk factors such as HIV co-infection, HBV co-infection, diabetes, metabolic dysfunction-associated steatohepatitis, or moderate to heavy alcohol use face a higher risk of HCC even after achieving SVR. These co-existing conditions are increasingly common, particularly due to the high prevalence of alcohol-associated liver disease and metabolic dysfunction-associated steatotic liver disease. The compounded risk of HCC in these patients makes annual US surveillance more justifiable, even in those with F3 fibrosis. However, a large randomized controlled study is needed to confirm the effectiveness of annual US surveillance in this group [25].

**Figure 1.**
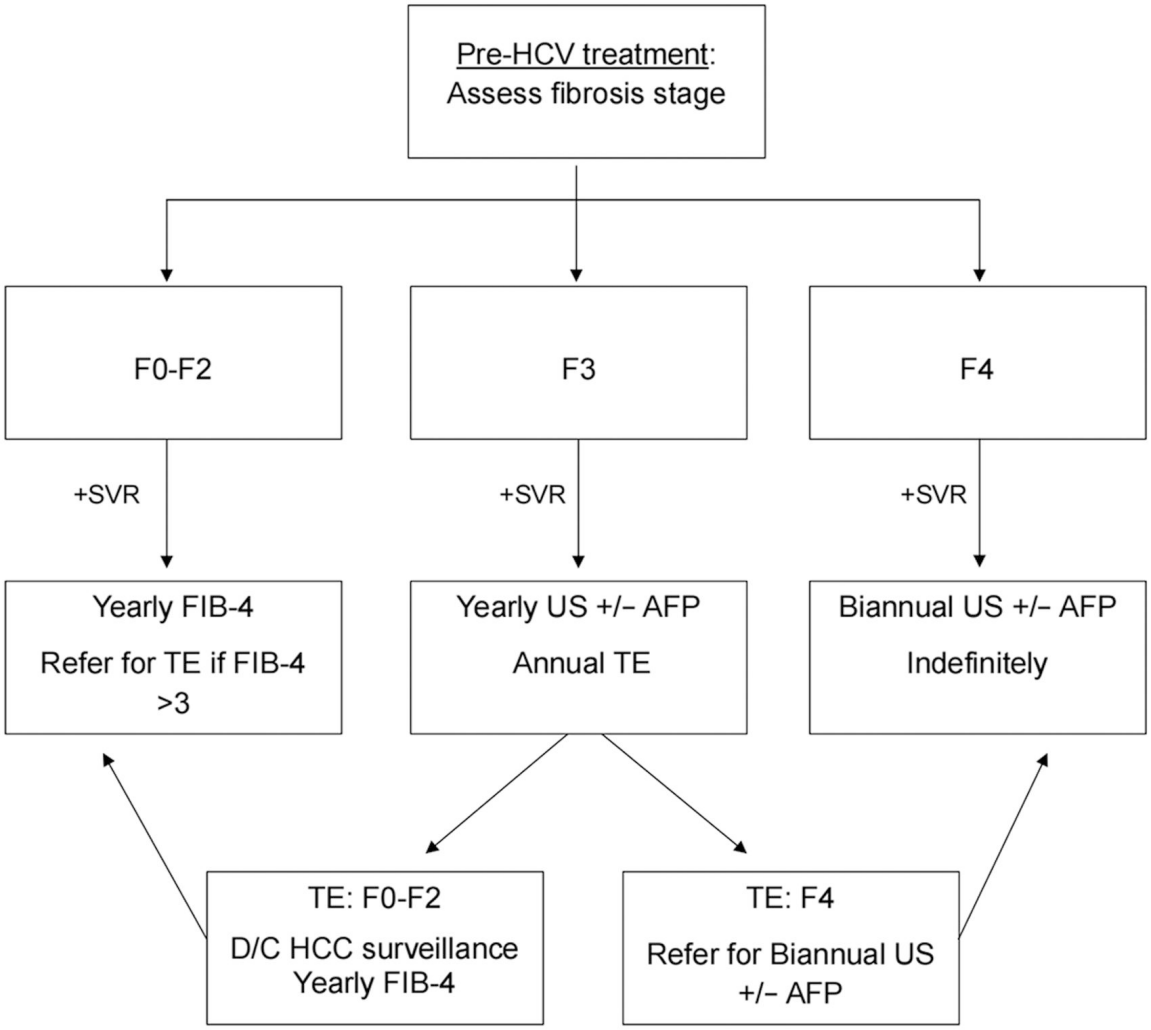
A simplified algorithm for the surveillance of hepatocellular carcinoma in patients who have achieved SVR after HCV treatment. The algorithm provides recommendations on the methods and interval of HCC surveillance based on the severity of pre-treatment fibrosis in patients with HCV who achieved SVR after DAA therapy.

The risk of HCC after achieving SVR appears to decline over time, likely due to the regression of fibrosis to below F3. Several studies suggest that as fibrosis regresses post-SVR, the annual risk of HCC decreases, particularly in patients without cirrhosis or in those whose fibrosis has regressed to F2 or lower [40]. Based on this evidence, we, the authors, recommend that all patients with compensated HCV cirrhosis who achieve SVR post-DAA therapy undergo semiannual AFP testing and US indefinitely. For patients with F3 fibrosis, we recommend annual US surveillance and TE. However, if fibrosis regresses to below F3, HCC surveillance can be discontinued in favor of annual FIB-4 assessments. This approach balances the need for ongoing surveillance in higher-risk patients with the importance of minimizing unnecessary procedures. For patients with SVR and F3 fibrosis who lack additional risk factors, continued annual AFP, US surveillance, and TE are recommended, but these can be discontinued if TE confirms regression to F2 or less, where the risk of HCC becomes minimal (Figure 1). This strategy reflects a more individualized, risk-based approach that considers both the benefits of surveillance and the potential costs and harms, tailored to the patient’s evolving risk profile [23].

## Conclusions

The introduction of DAAs has dramatically transformed the management of HCV, leading to a significant reduction in the disease burden. The observed decrease in HCC risk over time is likely associated with the gradual regression of liver fibrosis following HCV eradication. However, despite these advancements, the persistent risk of HCC among individuals with cirrhosis or advanced fibrosis (F3) after achieving SVR underscores the need for a strategic approach to HCC surveillance. Drawing from the evidence, the authors of the current review propose a simplified, cost-effective, and targeted surveillance strategy for patients who have achieved SVR (Figure 1). Further studies, including randomized clinical trials, are essential to validate and refine this surveillance algorithm.

## Authors’ Contributions

J.S.P. and C.Q.P. wrote the manuscript. A.J.P. assisted with the literature search, data extraction and collection, and composing tables and figures. All authors read and approved the final manuscript.

## Figures and Tables

**Table 1.** Recommendations from international guidelines on HCC surveillance after DAA therapy

Pre-DAA assessment	AASLD	EASL	APASL
F0–F2	N/A	N/A	US+tumor markers^a^ every 6 months for 2 years, followed by annually indefinitely
F3	No	US±AFP every 6 months indefinitely	US+tumor markers^a^ every 6 months indefinitely
F4	US±AFP every 6 months indefinitely	US±AFP every 6 months indefinitely	US+tumor markers^a^ every 6 months indefinitely

AASLD = American Association for the Study of Liver Diseases, EASL = European Association for the Study of Liver, APASL = Asian Pacific Association for the Study of Liver, SVR = sustained virologic response, DAA = direct acting antiviral agents,  TE = transient elastography, FIB-4 = Fibrosis-4 index, US = ultrasound, N/A = not available, AFP = alpha-fetoprotein, AFP-L3 = lens culinaris agglutinin-reactive AFP isoform, DCP = des gamma carboxy-prothrombin.

a Tumor markers: AFP, AFL-L3, DCP.

## References

[1] McGlynn KA , Petrick JL, El-Serag HB. Epidemiology of hepatocellular carcinoma. Hepatology 2021;73(Suppl 1):4–13. 10.1002/hep.31288 PMC7577946 32319693

[2] De Mitri MS , Poussin K, Baccarini P et al HCV-associated liver cancer without cirrhosis. Lancet 1995;345:413–5. 7772123 10.1016/s0140-6736(95)90400-x

[3] El-Serag HB , Kanwal F. Epidemiology of hepatocellular carcinoma in the United States: where are we? Where do we go? Hepatology 2014;60:1767–75. 24839253 10.1002/hep.27222 PMC4211957

[4] Dennis BB , Naji L, Jajarmi Y, Ahmed A, Kim D. New hope for hepatitis C virus: Summary of global epidemiologic changes and novel innovations over 20 years. World J Gastroenterol 2021;27:4818–30. 34447228 10.3748/wjg.v27.i29.4818 PMC8371499

[5] World Health Organization . Global Health Sector Strategy on Viral Hepatitis, 2016–2021. Geneva, Switzerland: World Health Organization; 2016. https://apps.who.int/iris/bitstream/handle/10665/246177/WHO-HIV-2016.06-eng.pdf (15 April 2024, date last accessed).

[6] Elsharkawy A , Samir R, El-Kassas M. Fibrosis regression following hepatitis C antiviral therapy. World J Hepatol 2022;14:1120–30. 35978676 10.4254/wjh.v14.i6.1120 PMC9258254

[7] Rockey DC , Friedman SL. Fibrosis regression after eradication of hepatitis C virus: from bench to bedside. Gastroenterology 2021;160:1502–20.e1. 33529675 10.1053/j.gastro.2020.09.065 PMC8601597

[8] Calvaruso V , Craxì A. Hepatic benefits of HCV cure. J Hepatol 2020;73:1548–56. 32777323 10.1016/j.jhep.2020.08.006

[9] Butt AA , Yan P, Shaikh OS et al Treatment of HCV reduces viral hepatitis-associated liver-related mortality in patients: an ERCHIVES study. J Hepatol 2020;73:277–84. 32145260 10.1016/j.jhep.2020.02.022

[10] Kanwal F , Kramer J, Asch SM et al Risk of hepatocellular cancer in HCV patients treated with direct-acting antiviral agents. Gastroenterology 2017;153:996–1005.e1. 28642197 10.1053/j.gastro.2017.06.012

[11] Waziry R , Hajarizadeh B, Grebely J et al Hepatocellular carcinoma risk following direct-acting antiviral HCV therapy: a systematic review, meta-analyses, and meta-regression. J Hepatol 2017;67:1204–12. 28802876 10.1016/j.jhep.2017.07.025

[12] Lok AS , Seeff LB, Morgan TR et al; HALT-C Trial Group. Incidence of hepatocellular carcinoma and associated risk factors in hepatitis C-related advanced liver disease. Gastroenterology 2009;136:138–48. 18848939 10.1053/j.gastro.2008.09.014 PMC3749922

[13] Ioannou GN , Green PK, Berry K. HCV eradication induced by DAA reduces the risk of hepatocellular carcinoma. J Hepatol 2018;68:25–32. 10.1016/j.jhep.2017.08.030 PMC5837901 28887168

[14] Carrat F , Fontaine H, Dorival C et al; French ANRS CO22 Hepather Cohort. Clinical outcomes in patients with chronic hepatitis C after direct-acting antiviral treatment: a prospective cohort study. Lancet 2019;393:1453–64. 30765123 10.1016/S0140-6736(18)32111-1

[15] Calvaruso V , Cabibbo G, Cacciola I et al; Rete Sicilia Selezione Terapia–HCV (RESIST-HCV). Incidence of hepatocellular carcinoma in patients with HCV-associated cirrhosis treated with direct-acting antiviral agents. Gastroenterology 2018;155:411–21.e4. 29655836 10.1053/j.gastro.2018.04.008

[16] Abu-Freha N , Abu-Kosh O, Yardeni D et al Liver fibrosis regression and associated factors in HCV patients treated with direct-acting antiviral agents. Life (Basel) 2023;13:1872. 37763276 10.3390/life13091872 PMC10533124

[17] Singh S , Fujii LL, Murad MH et al Liver stiffness is associated with risk of decompensated cirrhosis, hepatocellular carcinoma, and death in patients with chronic liver diseases: a systematic review and meta-analysis. Clin Gastroenterol Hepatol 2013;11:1573–84.e2. 23954643 10.1016/j.cgh.2013.07.034 PMC3900882

[18] Bhattacharya D , Aronsohn A, Price J et al; AASLD-IDSA HCV Guidance Panel. Hepatitis C Guidance 2023 Update: AASLD-IDSA recommendations for testing, managing, and treating hepatitis C virus infection. Clin Infect Dis 2023:ciad319. 10.1093/cid/ciad319 37229695

[19] Vallet-Pichard A , Mallet V, Nalpas B et al FIB-4: an inexpensive and accurate marker of fibrosis in HCV infection. Comparison with liver biopsy and fibrotest. Hepatology 2007;46:32–6. 17567829 10.1002/hep.21669

[20] Sterling RK , Lissen E, Clumeck N et al; APRICOT Clinical Investigators. Development of a simple noninvasive index to predict significant fibrosis patients with HIV/HCV co-infection. Hepatology 2006;43:1317–25. 16729309 10.1002/hep.21178

[21] Ioannou GN, BesteLA, GreenPK et al Increased risk for hepatocellular carcinoma persists up to 10 years after HCV eradication in patients with baseline cirrhosis or high FIB-4 scores. *Gastroenterology* 2019;157:1264–78.e4. 10.1053/j.gastro.2019.07.033 PMC6815714 31356807

[22] Tapper EB , LokAS. Use of liver imaging and biopsy in clinical practice. N Engl J Med 2017;377:756–68. 28834467 10.1056/NEJMra1610570

[23] You MW , Kim KW, Shim JJ et al Impact of liver-stiffness measurement on hepatocellular carcinoma development in chronic hepatitis C patients treated with direct-acting antivirals: a systematic review and time-to-event meta-analysis. J Gastroenterol Hepatol 2021;36:601–8. 32875681 10.1111/jgh.15243

[24] Ogasawara N , Saitoh S, Akuta N et al Advantage of liver stiffness measurement before and after direct-acting antiviral therapy to predict hepatocellular carcinoma and exacerbation of esophageal varices in chronic hepatitis C. Hepatol Res 2020;50:426–38. 31785120 10.1111/hepr.13467

[25] Degasperi E , D’Ambrosio R, Iavarone M et al Factors associated with increased risk of de novo or recurrent hepatocellular carcinoma in patients with cirrhosis treated with direct-acting antivirals for HCV infection. Clin Gastroenterol Hepatol 2019;17:1183–91.e7. 30613002 10.1016/j.cgh.2018.10.038

[26] El-Serag HB , Kanwal F, Richardson P et al Risk of hepatocellular carcinoma after sustained virological response in veterans with hepatitis C virus infection. Hepatology 2016;64:130–7. 26946190 10.1002/hep.28535 PMC4917456

[27] Nahon P , Layese R, Bourcier V et al; ANRS CO12 CirVir Group. Incidence of hepatocellular carcinoma after direct antiviral therapy for HCV in patients with cirrhosis included in surveillance programs. Gastroenterology 2018;155:1436–50.e6. 30031138 10.1053/j.gastro.2018.07.015

[28] Marrero JA , Fontana RJ, Fu S et al Alcohol, tobacco and obesity are synergistic risk factors for hepatocellular carcinoma. J Hepatol 2005;42:218–24. 15664247 10.1016/j.jhep.2004.10.005

[29] Galle PR , Forner A, Llovet JM et al EASL Clinical Practice Guidelines: management of hepatocellular carcinoma. J Hepatol 2018;69:182–236. 29628281 10.1016/j.jhep.2018.03.019

[30] Marrero JA , Kulik LM, Sirlin CB et al Diagnosis, staging, and management of hepatocellular carcinoma: 2018 Practice Guidance by the American Association for the Study of Liver Diseases. Hepatology 2018;68:723–50. 29624699 10.1002/hep.29913

[31] Allaire M , Bruix J, Korenjak M et al What to do about hepatocellular carcinoma: recommendations for health authorities from the International Liver Cancer Association. J Hepathol Rep 2022;4:100578. 10.1016/j.jhepr.2022.100578 PMC9638834 36352896

[32] Kanda T , Lau GKK, Wei L et al APASL HCV guidelines of virus-eradicated patients by DAA on how to monitor HCC occurrence and HBV reactivation. Hepatol Int 2019;13:649–61. 31541423 10.1007/s12072-019-09988-7 PMC6861433

[33] Llovet JM , Kelley RK, Villanueva A et al Hepatocellular carcinoma. Nat Rev Dis Primers 2021;7:6. Erratum in: *Nat Rev Dis Primers* 2024;10:10. 33479224 10.1038/s41572-020-00240-3 PMC13537433

[34] Lin OS , Keeffe EB, Sanders GD et al Cost-effectiveness of screening for hepatocellular carcinoma in patients with cirrhosis due to chronic hepatitis C. Aliment Pharmacol Ther 2004;19:1159–72. 15153169 10.1111/j.1365-2036.2004.01963.x

[35] Arguedas MR , Chen VK, Eloubeidi MA et al Screening for hepatocellular carcinoma in patients with hepatitis C cirrhosis: a cost-utility analysis. Am J Gastroenterol 2003;98:679–90. 12650806 10.1111/j.1572-0241.2003.07327.x

[36] Farhang Zangneh H , Wong WWL, Sander B et al Cost effectiveness of hepatocellular carcinoma surveillance after a sustained virologic response to therapy in patients with hepatitis C virus infection and advanced fibrosis. Clin Gastroenterol Hepatol 2019;17:1840–9.e16. 30580095 10.1016/j.cgh.2018.12.018

[37] Yoon JH , Lee JM, Lee DH et al A comparison of biannual two-phase low-dose liver CT and US for HCC surveillance in a group at high risk of HCC development. Liver Cancer 2020;9:503–17. 33083277 10.1159/000506834 PMC7548851

[38] Gupta P , Soundararajan R, Patel A et al Abbreviated MRI for hepatocellular carcinoma screening: a systematic review and meta-analysis. J Hepatol 2021;75:108–19. 33548385 10.1016/j.jhep.2021.01.041

[39] Lockart I , Yeo MGH, Hajarizadeh B et al HCC incidence after hepatitis C cure among patients with advanced fibrosis or cirrhosis: a meta-analysis. Hepatology 2022;76:139–54. 35030279 10.1002/hep.32341 PMC9303770

[40] Semmler G , Meyer EL, Kozbial K et al HCC risk stratification after cure of hepatitis C in patients with compensated advanced chronic liver disease. J Hepatol 2022;76:812–21. 34871626 10.1016/j.jhep.2021.11.025

